# Editorial: Functional Foods and Bioactive Food Ingredients in Prevention and Alleviation of Metabolic Syndrome

**DOI:** 10.3389/fnut.2021.788941

**Published:** 2021-10-29

**Authors:** Sebastian Torres, Roxana Beatriz Medina, Maria Isabel Vasallo Morillas, María Inés Isla, Paola Gauffin-Cano

**Affiliations:** ^1^Instituto de Bioprospección y Fisiología Vegetal (INBIOFIV, CONICET - UNT), San Miguel de Tucumán, Argentina; ^2^Facultad de Ciencias Naturales e IML, UNT, San Miguel de Tucumán, Argentina; ^3^Centro de Referencia para Lactobacilos (CERELA, CONICET), San Miguel de Tucumán, Argentina; ^4^San Antonio Technologies, Catholic University San Antonio of Murcia, Murcia, Spain

**Keywords:** functional foods, bioactive compounds, metabolic syndrome, obesity, gut microbiome, adipose tissue, chronic inflammation, oxidative stress

Metabolic syndrome (MetS) is a global metabolic disorder characterized by a constellation of interconnected risk factors, including central obesity (increased waist circumference), dysregulation of glucose (insulin resistance and hyperglycemia), hypertension, and dyslipidemia (hypertriglyceridemia and reduced HDL levels) (1). Despite this clinical condition occur when three or more of these metabolic risk factors are present, abdominal obesity is a common denominator in MetS (1, 2). The excess of visceral adiposity is a source of pro-inflammatory adipokines that contributes to the prevalence of chronic low-grade inflammation characteristic of MetS (1). Thus, expansion of abdominal adipose tissue can lead to oxidative stress, systemic inflammation, insulin resistance, and endothelial damage. The perpetuation of these dysfunctions, co-occurring with other metabolic derangements, can eventually develop into cardiovascular diseases, type 2 diabetes mellitus, non-alcoholic fatty liver disease, some obesity-related cancers, and cause early death (1, 3). MetS is associated with several factors, including genetics, lifestyle, and chronic stress, among others, which favors dysregulation of energy balance, promoting fat accumulation (4, 5). Chiefly, unhealthy lifestyles (physical inactivity, sleep disorders, stress mismanagement, Western dietary patterns, and smoking and drinking habits) have boosted the quick progress of the MetS. As a result, in this early twenty-first century, MetS incidence and prevalence have unrestraint increase, and this clinical condition has become a significant public health issue worldwide (2, 4). It is estimated that the global prevalence of MetS is around 25% of the world population (over a billion people worldwide); however, this percentage increases in the adult population over 40 years old (4, 6).

The main therapeutic guideline applied in the case of MetS is aimed at modifying lifestyle, with calorie restriction as primary treatment (7). Indeed, lifestyle intervention can be effective in reducing the prevalence of MetS as well as the severity of its metabolic risk factors (8). In this sense, recent systematic reviews and meta-analyses have demonstrated that supervised multifaceted lifestyle modification, including at least diet combined with physical activity, can improve MetS and its clinical implications (8). However, often MetS patients require pharmaceutical therapy, frequently using multiple drugs, which generally cause side effects. It is in particular in these cases where natural bioactive compounds come into play. Functional foods and bioactive food ingredients emerge as potential prophylactic and therapeutic alternatives for MetS and its typical comorbidities. They represent versatile tools for addressing different aspects of this syndrome, directly or indirectly restoring the equilibrium of gut microbiota, gut barrier function, energy homeostasis, immune function, and redox balance (2, 9).

In the Research Topic *Functional foods and bioactive food ingredients in prevention and alleviation of metabolic syndrome*, these multiple talents of functional foods and bioactive food compounds are addressed, focusing on the effects of biofunctional dietary factors in the past two decades (Cao et al.), antioxidant vitamin C on MetS (Guo et al.), green tea compounds on browning process of adipocytes (Otton et al.), soy protein on hepatic Cytochrome P450 expression (Kozaczek et al.), vegetables of the Cruciferous family on metabolic parameters and gut microbiome composition (Zandani et al.), and probiotics on metabolic and immunological parameters and gut microbiota composition (Fabersani et al.).

In the investigation by Cao et al. the authors reviewed the research status of dietary factors associated with MetS by searching relevant studies on the field in The Web of Science database from 2000 to 2020. Through the bibliometric analysis of data and the biclustering analysis of word co-occurrence (gCLUTO software), the social network analysis of highly-frequent keywords and highly cited papers (Ucinet software), and the theme evolutionary analysis of research themes (SciMAT software), the authors systematically mapped the research hotspots, the knowledge structure, and the theme trends of this field in the past two decades. The thorough analysis made by Cao et al. identified the fatty acids, dietary fiber, and polyphenols in the focus of research on the diet components related to MetS over the years, highlighting fish oil and vitamin C as recently developed Research Topics. In addition, authors recognized prebiotics as an emerging theme with developmental potential.

The association of vitamin C, a research trend identified by Cao et al. with MetS was studied by Guo et al.. The authors carried up a meta-analysis of 28 observational studies regarding the associations of dietary and circulating vitamin C levels with MetS. This review concluded that both dietary and circulating vitamin C levels are inversely associated with MetS. Further, the scientific evidence supports the potential beneficial effect of vitamin C on this syndrome, probably relying on its capacity to scavenge harmful free radicals and promote neutrophils clearance by macrophages.

A reasonable strategy to ameliorate obesity aims to enhance energy expenditure by increasing adipose tissue thermogenesis (through the browning of adipocytes) using natural bioactive compounds. In this context, Otton et al. analyzed the ability of green tea compounds to induce (autonomously) thermogenic recruitment in adipocytes. Previous studies have demonstrated that green tea compounds can increase energy expenditure. Contrary to what was previously observed in adipogenic cell lines, Otton et al. showed a negative effect of green tea compounds on basal UCP1 (uncoupling protein 1) gene expression in both brown and white primary adipocytes. According to this observation, the results obtained in adipogenic cell lines cannot unreservedly be extrapolated to authentic brown and white adipocytes.

Non-alcoholic fatty liver disease (NAFLD), a pathology characterized by fat accumulation and inflammation in the liver tissue, is strongly associated with MetS. Cytochrome P450 (CYP450) superfamily, which are the main detoxifying enzymes in the liver, are differentially expressed in the liver throughout the progression of NAFLD. In their research, Kozaczek et al. evaluated the effect of a soy protein diet in an animal model of NAFLD. The authors found that the expression of CYP450 genes was modified after a soy-based diet and that this change could result in the attenuation of liver steatosis.

In another approach to intervening the NAFLD, Zandani et al. explored the impact of whole broccoli supplementation (florets and stalks) on metabolic parameters, liver histology, and gut microbiome composition of high-fat diet-fed mice. They observed that broccoli florets improved glucose homeostasis and upregulated adiponectin receptor expression. Besides, both broccoli stalks and florets altered the diversity and structure of the intestinal microbiota environment to favor inflammatory status and integrity of the gut.

Also, the modulation of gut microbiota together with the adjustment of the function of adipose tissue was the focus of the research of Fabersani et al. The authors evaluated the effect of probiotic lactic acid bacteria with proved adipo- and immuno-modulatory capacities on metabolic/immunological parameters and composition of gut microbiota in mice fed a high-fat diet. They found that tested probiotic strains differentially modulated the intestinal microbiota and improved the evaluated parameters, highlighting two bacterial strains as potential adjuvants for nutritional treatment of obesity and overweight.

The current Research Topic constitutes a sample of the wide variety of alternatives to prevent or improve MetS and its comorbidities by using functional foods or bioactive ingredients, as well so the trends in Research Topics in the field and the new methodologies to approach the study of the associations between bioactive compounds and the metabolic disorders. Overall, further elucidation of the bioactive compounds responsible for the described results is required to deepen the underlying mechanisms by which functional foods and their functional ingredients face MetS.

## Author Contributions

All authors listed have made a substantial, direct and intellectual contribution to the work, and approved it for publication.

## Funding

This study was supported by a CONICET Grant PIP (No. 215) and by an ANPCyT- FONCyT Grant PICT2017 (No. 3975) from Argentina.

## Conflict of Interest

The authors declare that the research was conducted in the absence of any commercial or financial relationships that could be construed as a potential conflict of interest.

## Publisher's Note

All claims expressed in this article are solely those of the authors and do not necessarily represent those of their affiliated organizations, or those of the publisher, the editors and the reviewers. Any product that may be evaluated in this article, or claim that may be made by its manufacturer, is not guaranteed or endorsed by the publisher.
